# *Faecalibacterium prausnitzii* in neurological disorders: mechanistic links and therapeutic potential along the microbiota–gut–brain axis

**DOI:** 10.3389/fmicb.2026.1962324

**Published:** 2026-10-23

**Authors:** Yuan Liu, Guangping Yang, Lang Su, Hong Wang, Qianhui Zhou, Peng Yao, Can Jing, Yang Bai, Zhen Feng

**Affiliations:** 1 Affiliated Rehabilitation Hospital, Jiang Xi Medical College, Nanchang University, Nanchang, Jiangxi, China; 2 Rehabilitation Medicine Clinical Research Center of Jiangxi Province, Nanchang, Jiangxi, China; 3 Key Laboratory of Jiangxi Provincial Health Commission for DOC Rehabilitation, Nanchang, Jiangxi, China

**Keywords:** *Faecalibacterium prausnitzii*, gut microbiota, host health, neurological diseases, probiotics

## Abstract

Widespread dysbiosis in neurological disorders highlights the critical role of the microbiota–gut–brain axis (MGBA) and the therapeutic potential of probiotics in modulating the central nervous system (CNS). *Faecalibacterium prausnitzii*, a key butyrate-producing commensal bacterium in the human gut, has attracted broad attention as a candidate next-generation probiotic (NGP). This review aims to describe and discuss the mechanisms of action of *F. prausnitzii* and its potential in the prevention and management of neurological disorders. Multiple human observational cohort studies indicate that the relative abundance of *F. prausnitzii*—or the genus *Faecalibacterium*—is negatively correlated with disease severity in conditions such as Alzheimer’s disease (AD), Parkinson’s disease (PD), stroke, and multiple sclerosis (MS); however, the directionality of this association remains discordant in neurodevelopmental conditions such as autism spectrum disorder (ASD). To date, interventional evidence is predominantly derived from rodent models, in which oral gavage of specific *F. prausnitzii* strains or synbiotic formulations has been reported to alleviate cognitive deficits, motor dysfunction, anxiety-like behaviors, and pathological protein deposition. Mechanistically, these neuroprotective effects involve diverse *F. prausnitzii*-derived neuroactive metabolites—including the microbial anti-inflammatory molecule (MAM), extracellular vesicles (EVs), and phenolic derivatives—as well as indirect pathways mediated by butyrate through histone deacetylase (HDAC) inhibition and intestinal barrier fortification. Given the current lack of clinical intervention trials directly administering live *F. prausnitzii* in human neurological populations, future research requires rigorously designed, strain-specific randomized controlled trials (RCTs) to establish its clinical safety, colonization efficiency, and therapeutic efficacy.

## 1 Introduction

Neurological disorders represent a major global public health challenge. According to the Global Burden of Disease (GBD) 2021 study, an umbrella of 37 conditions affecting the nervous system—predominantly driven by highly prevalent disorders such as tension-type headaches, migraine, and stroke, alongside progressive neurodegenerative pathologies—collectively ranks as the leading cause of disability-adjusted life years (DALYs) globally, affecting an estimated 3.40 billion individuals(G.B.D.N.S.D. Collaborators, 2024). With an aging global population, the prevalence of neurodegenerative diseases and associated functional impairments is expected to rise further, placing a severe strain on healthcare systems worldwide (Cao et al., 2025). Conventional central nervous system (CNS) pharmacotherapies face significant bottlenecks, including poor blood–brain barrier (BBB) penetration, systemic toxicity, and adverse effects from long-term use. Consequently, there is an urgent need for novel intervention strategies that combine biocompatibility with multi-target regulatory capacity (Persson et al., 2026; Li et al., 2024).

The discovery of the microbiota–gut–brain axis (MGBA) has provided a fresh perspective for neuroscience research (Zhang et al., 2025). Characteristic alterations in microbial profiles and gut dysbiosis are widely observed in patients with neurodevelopmental and neurodegenerative disorders (Hawkins et al., 2025; Warren et al., 2026). Dysbiosis profoundly alters CNS homeostasis by triggering systemic inflammation, modulating neurotransmitter synthesis, and impairing intestinal barrier permeability (Khan et al., 2025). Restoring gut microbiome homeostasis to modulate host neurochemical processes has therefore become a pivotal strategy for developing alternative therapeutics (Schneider et al., 2024). Currently, interventions such as fecal microbiota transplantation (FMT), prebiotics, and probiotic supplementation are attracting broad academic attention (Sun et al., 2020; Rungsri et al., 2017; Kim et al., 2024; Angelucci et al., 2019). Given the lack of established clinical protocols for many major neurological diseases, elucidating the underlying mechanisms and developing emerging therapeutic strategies have become imperative in contemporary neuroscience (Zhou et al., 2023).

Within the complex gut ecosystem, *Faecalibacterium prausnitzii* (*F. prausnitzii*) is recognized as one of the most vital commensal species for human health and serves as a key biomarker of intestinal homeostasis (Effendi et al., 2022). As a representative butyrate-producing bacterium of the phylum Firmicutes, *F. prausnitzii* plays a crucial role in maintaining intestinal barrier integrity and modulating systemic immune homeostasis (Lopez-Siles et al., 2017; Avram-Hananel et al., 2010). Reduced abundance of *F. prausnitzii* has been implicated in the pathogenesis of various inflammatory, metabolic, and autoimmune diseases (He et al., 2020; Fujimura et al., 2016; Song et al., 2016; Eppinga et al., 2016). Due to its robust anti-inflammatory properties, *F. prausnitzii* has been proposed as a promising next-generation probiotic (NGP) candidate, although its relevance to neurological disorders requires further clinical validation (Al-Fakhrany and Elekhnawy, 2024). However, research on *F. prausnitzii* has largely focused on intestinal inflammation, and its progress in neurological diseases has not been comprehensively reviewed. To our knowledge, this is the first review to specifically and comprehensively examine the role of *F. prausnitzii* in neurological and neuropsychiatric disorders. By integrating human observational evidence, strain-specific preclinical findings, and potential mechanisms along the MGBA, we further distinguish species-linked effects from shared microbial metabolic pathways and associative findings from causal evidence.

### 1.1 Literature search strategy

As a narrative review, we performed a structured literature search in PubMed and Web of Science from database inception to September 2026. Search terms combined “*Faecalibacterium prausnitzii*” or “Faecalibacterium” with terms related to the MGBA and the neurological or neuropsychiatric disorders covered in this review. Original human and preclinical studies reporting *F. prausnitzii* or *Faecalibacterium* abundance, mechanistic pathways, or intervention-related outcomes were prioritized, whereas studies without relevant neurological outcomes or separable *F. prausnitzii/Faecalibacterium* information were excluded.

## 2 Characteristics of *F. prausnitzii*

*F. prausnitzii*, formerly known as *Fusobacterium prausnitzii*, is a rod-shaped, non-spore-forming, non-motile, Gram-positive obligate anaerobe belonging to the phylum *Firmicutes* (Louis and Flint, 2009). Typically lacking plasmids, *F. prausnitzii* possesses a circular chromosome of approximately 2.93–3.32 Mb, predicted to encode roughly 3,000 proteins, with a relatively low guanine-cytosine content of 47–57% (Foditsch et al., 2014; Lopez-Siles et al., 2012). *F. prausnitzii* exhibits mesophilic growth and extreme oxygen sensitivity, requiring stringent conditions for *in vitro* culture. These characteristics reflect its specialized adaptation to the anaerobic niche of the gut (Louis and Flint, 2009; Duncan et al., 2002). This bacterium is a dominant member of *Clostridium cluster IV* in the human colon and represents the second most abundant species identified in human fecal samples (Duncan and Flint, 2008). In healthy adult gut microbiota, *F. prausnitzii* typically accounts for over 5% of total bacteria, reaching up to 15% in some individuals (Castillo et al., 2007; Oikonomou et al., 2013; Martin et al., 2017). It is also widely distributed across the gastrointestinal tracts of various mammals, including pigs and cattle. The high abundance and broad distribution of *F. prausnitzii* highlight its role as a core member of the gut microbiota with profound impacts on host physiology and health (Effendi et al., 2022; Zheng et al., 2025).

As sequencing technologies have evolved from 16S rRNA analysis to whole-genome sequencing, *F. prausnitzii* has demonstrated remarkable intra-species diversity (Miquel et al., 2013). Historically, this species was classified into two main phylogroups: Phylogroup I (represented by ATCC 27768) and Phylogroup II (represented by A2-165) (Lopez-Siles et al., 2012). Recent genomic studies have revealed additional phylogenetic clades with distinct metabolic features. Among these, Clade A, represented by the UT1 strain, shows a high prevalence in global populations despite being long overlooked (Geng et al., 2025). Genomic annotation reveals that UT1 harbors a unique set of carbohydrate-active enzymes, including glycoside hydrolases, polysaccharide lyases, and carbohydrate esterases (Geng et al., 2025). Compared with the classic strain A2-165, UT1 demonstrates superior ecological remodeling capacity in modulating the intestinal microenvironment. Oral administration of UT1 significantly enriches intestinal *Lactobacillus* and *Bifidobacterium* populations (Geng et al., 2025). This inter-species facilitation enhances the stability of the gut metabolic network. Such microbiome remodeling may indirectly influence host metabolic and immune signaling; however, whether these strain-specific ecological effects translate into altered brain function remains to be established (Socala et al., 2021).

## 3 Potential mechanisms linking *F. prausnitzii* to neurological and neuropsychiatric disorders

*F. prausnitzii* is recognized as a promising next-generation probiotic (NGP) because of its roles in immune regulation, gut homeostasis, and systemic metabolism (Effendi et al., 2022). However, direct evidence linking *F. prausnitzii* to neurological disorders remains limited, and most human studies report associations between its abundance and disease-related features rather than establishing causality. Several potential mechanisms have nevertheless been proposed, including the production of neuroactive metabolites, preservation of intestinal barrier integrity, and modulation of host immune responses (Figure 1). Importantly, species-linked effects should be distinguished from shared microbial metabolic pathways. *F. prausnitzii*-derived factors such as the microbial anti-inflammatory molecule (MAM), extracellular vesicles (EVs), and phenolic derivatives provide relatively species-linked evidence, whereas butyrate-mediated effects are less specific because butyrate is produced by multiple anaerobic bacteria. Therefore, butyrate/SCFA signaling, broader microbiome shifts, and multistrain interventions should be interpreted as indirect or non-specific evidence rather than effects unique to *F. prausnitzii*.

**Figure 1:**
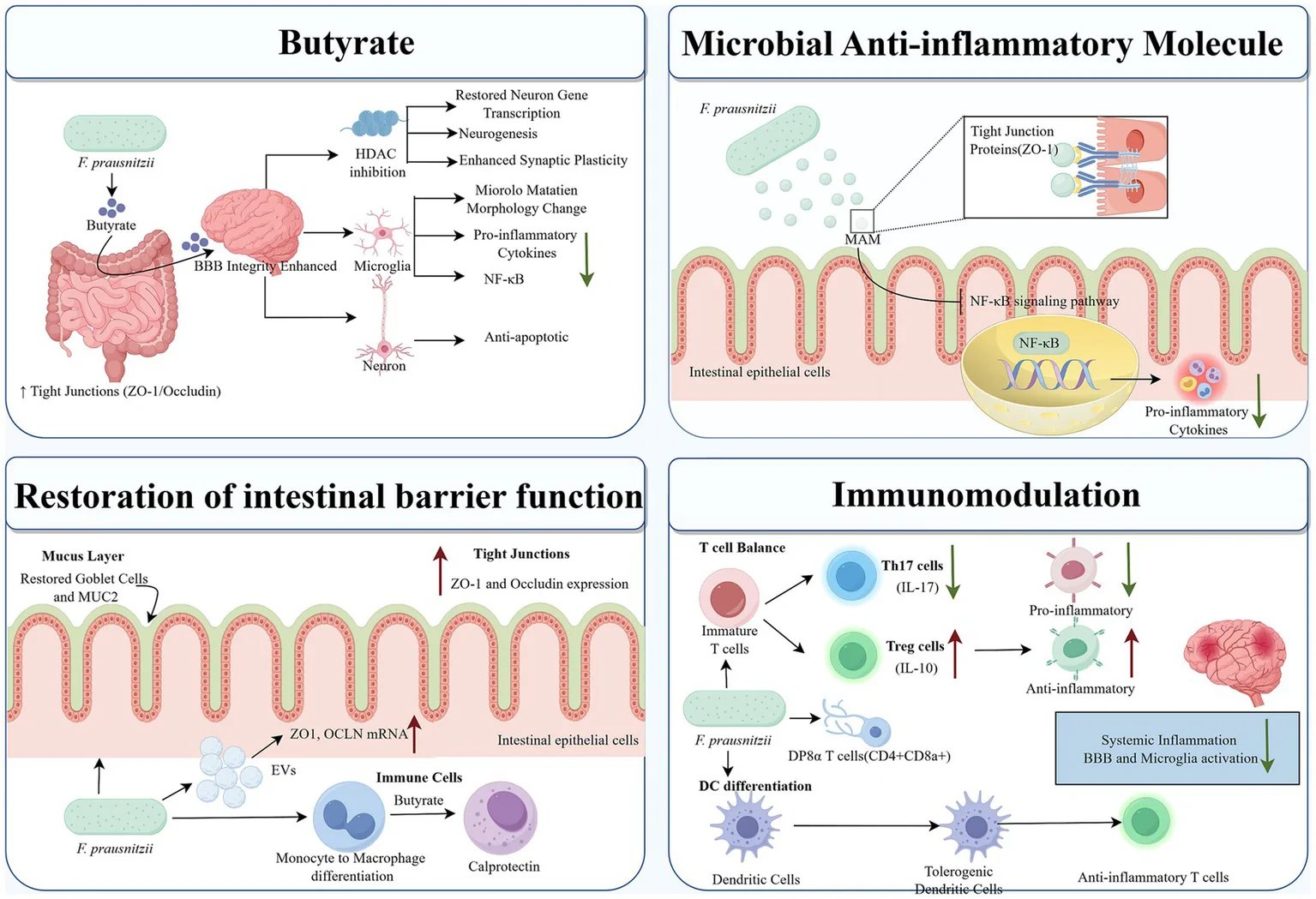
Multifaceted mechanisms by which *F. prausnitzii* protects the central nervous system via the microbiota–gut–brain axis. *Faecalibacterium prausnitzii* exerts neuroprotective effects through multiple mechanisms: (1) producing neuroactive metabolites such as butyrate, (2) secreting microbial anti-inflammatory molecule (MAM), (3) restoring intestinal barrier structure and function, and (4) modulating host immune-inflammatory responses. BBB, Blood–brain barrier; HDAC, Histone deacetylase; NF-κB, Nuclear factor-kappa B; ZO-1, Zonula occludens-1; MAM, Microbial anti-inflammatory molecule; MUC2, Mucin 2; EVs, Extracellular vesicles; ZO1, Zonula occludens-1 gene; OCLN, Occludin gene; Th17, T helper 17; Treg, Regulatory T; IL-17, Interleukin-17; IL-10, Interleukin-10; DP8α, Double-positive CD4 + CD8α+; DC, Dendritic cell.

### 3.1 Neuroactive metabolites

The biological effects of *F. prausnitzii* are largely driven by its diverse metabolic output. Multiple studies indicate that colonization by *F. prausnitzii* significantly reshapes host metabolic profiles, enriching key pathways such as synaptic vesicle cycling and neuroactive ligand–receptor interactions. These findings support several candidate metabolic pathways through which *F. prausnitzii* may influence host physiology. However, for most metabolites, direct transport from *F. prausnitzii* in the gut to the CNS and subsequent species-specific effects on neurological outcomes have not been established. Therefore, these pathways should primarily be considered biologically plausible mechanisms rather than direct evidence of CNS delivery.

#### 3.1.1 Short-chain fatty acids: butyrate

The SCFA butyrate is a primary metabolic product of *F. prausnitzii* (Zhang et al., 2025). Within the intestinal niche, *F. prausnitzii* displays strong ecological cross-feeding, efficiently converting acetate produced by primary degraders such as *Bifidobacterium* into butyrate (Louis and Flint, 2009; Moens et al., 2016; Rios-Covian et al., 2015). Butyrate provides approximately 70% of the energy consumed by colonocytes and efficiently crosses the blood–brain barrier (BBB) to enter the CNS (Gasaly et al., 2021; Silveira et al., 2023). Subsequently, butyrate stimulates the AKT/AMPK signaling pathway to upregulate tight junction proteins, reinforcing both intestinal and BBB integrity. This protective mechanism effectively prevents pro-inflammatory cytokines and cytotoxic metabolites from penetrating the central nervous system (Silveira et al., 2023; Peng et al., 2009; Wang et al., 2012; Canani et al., 2011). Upon crossing the BBB, butyrate exerts epigenetic regulation through its potent histone deacetylase (HDAC) inhibitory activity. By inducing histone acetylation, butyrate restores impaired neuronal gene transcription, promotes neurogenesis, and enhances synaptic plasticity (Xu et al., 2026; Jaworska et al., 2019; Yoo et al., 2011). In an APP/PS1-21 mouse model of Alzheimer’s disease (AD), butyrate restored hippocampal histone acetylation levels and effectively alleviated cognitive deficits (Govindarajan et al., 2011). In Parkinson’s disease (PD) models, it significantly elevated histone H3 acetylation in striatal tissues, protecting dopaminergic neurons and preventing toxin-induced motor impairments (Sharma et al., 2015). This multi-target transcriptional regulation endows butyrate with unique advantages in intervening in complex neuropathological processes. Butyrate also demonstrates marked immunomodulatory potential in neuroinflammation. Multiple studies have shown that it suppresses NF-κB signaling activation, thereby downregulating pro-inflammatory cytokines such as IL-18, IL-6, and TNF-α (Dang et al., 2021; Sun et al., 2025; Vinolo et al., 2011). Additionally, it promotes microglial maturation and morphological transitions in the brain, maintaining cellular homeostasis and strengthening central immune defense (Wang et al., 2018). Furthermore, butyrate regulates apoptotic balance by increasing the *Bcl-2/Bax* expression ratio, thereby inhibiting neuronal apoptosis and delaying neurodegenerative progression (Sun et al., 2015; Bayazid et al., 2021). Butyrate indirectly modulates brain function via enteroendocrine pathways. It stimulates intestinal L-cells to secrete glucagon-like peptide-1 (GLP-1) and peptide YY (PYY), which transmit metabolic signals to the CNS (Khan et al., 2025; Hirdaramani et al., 2026). In summary, butyrate represents one plausible mediator linking *F. prausnitzii* to the MGBA, with established roles in maintaining gut barrier integrity and colonocyte energy supply and potential effects on CNS function through epigenetic, immunomodulatory, and neuroendocrine pathways. Nevertheless, because these effects are not specific to *F. prausnitzii*, direct species- and strain-specific intervention studies are required to determine the extent to which *F. prausnitzii*-derived butyrate contributes to neurological outcomes.

#### 3.1.2 Microbial anti-inflammatory molecule

*F. prausnitzii* secretes a unique 15 kDa protein that assembles into an ordered lattice structure on the bacterial surface. Known as the MAM, it is one of the most abundant proteins in the bacterial envelope (Auger et al., 2022; Breyner et al., 2017; Quevrain et al., 2016). Its primary function is to suppress NF-κB signaling in host intestinal epithelial cells, thereby reducing pro-inflammatory cytokine production and directly attenuating local inflammation (Breyner et al., 2017). In addition, MAM plays a vital role in maintaining intestinal barrier integrity (Xu et al., 2020). It potentially interacts with tight junction proteins in the intestinal epithelium to reinforce barrier function (Xu et al., 2020). In a diabetic mouse model, Xu et al. observed reduced *F. prausnitzii* abundance. Supplementation with recombinant MAM protein significantly restored intestinal barrier function and upregulated ZO-1 expression. Co-immunoprecipitation further revealed potential interactions between MAM and key tight junction proteins, including ZO-1 (Xu et al., 2020). Through these dual mechanisms, MAM plays a crucial role in maintaining local intestinal immune homeostasis.

#### 3.1.3 Phenolic compounds: salicylic acid and shikimic acid

During metabolism, *F. prausnitzii* synthesizes anti-inflammatory phenolic compounds via the shikimate pathway, primarily producing shikimic acid and salicylic acid (Miquel et al., 2015). In a mouse model of acute colitis, Miquel et al. demonstrated that *F. prausnitzii* colonization significantly increased colon and serum levels of shikimic acid and salicylic acid. This increase was accompanied by reduced serum levels of pro-inflammatory cytokines, including IL-1α, IL-2, and IL-5 (Miquel et al., 2015). Shikimic acid regulates microglial activation, effectively suppressing lipopolysaccharide-induced overproduction of pro-inflammatory mediators and reactive oxygen species in BV-2 cells (Bao et al., 2023). Mechanistic studies further confirm that shikimic acid exerts antioxidant and neuroprotective effects by activating the AKT/Nrf2 pathway while concurrently inhibiting the NF-κB pathway (Bao et al., 2023). In a mouse model of Parkinson’s disease, shikimic acid was also shown to attenuate neurological damage and behavioral deficits (Bao et al., 2023). Meanwhile, salicylic acid may exert direct neuromodulatory effects by regulating the central GABAergic system (Motaghi et al., 2022). Motaghi et al. demonstrated that salicylic acid significantly upregulates GAD1 expression in the rat ventral hippocampus, exhibiting distinct anxiolytic, sedative, and hypnotic efficacy (Motaghi et al., 2022). However, evidence regarding the specific metabolic pathways producing these phenolic compounds in *F. prausnitzii* and their functions within the MGBA remains limited, requiring further validation.

### 3.2 Restoration of intestinal barrier function

Dysfunction of the intestinal mucosal barrier is deeply involved in the pathological progression of various neurological disorders, such as AD and PD (Fabi, 2024; Di Vincenzo et al., 2024). As a critical interface between gastrointestinal microbiota and host tissues, the intestinal mucosal barrier plays a central regulatory role in maintaining host-commensal homeostasis (Di Vincenzo et al., 2024; Mou et al., 2022; Parker et al., 2020). Preserving intestinal epithelial barrier integrity is essential for host health and neuroimmune homeostasis, constituting the primary defense against pathogen invasion and environmental stress (Turner, 2009; Okumura and Takeda, 2017). *F. prausnitzii* protects the intestinal barrier through multifaceted mechanisms (Zhang et al., 2021; Ferreira-Halder et al., 2017; Carlsson et al., 2013). It upregulates the expression of occludin and zonula occludens-1 (ZO-1) via direct and indirect pathways, strengthening the tight junction scaffold between intestinal epithelial cells and directly modulating mucosal permeability (Xu et al., 2020; Moosavi et al., 2020). For example, its secreted MAM efficiently blocks nuclear factor-kappa B (NF-κB) signaling activation. This protects the intestinal mucosa from immune-mediated structural damage and promotes barrier repair (Breyner et al., 2017; Xu et al., 2020). Additionally, *F. prausnitzii*-derived extracellular vesicles (EVs) act as long-range communication tools. They cross the mucus layer to directly target intestinal epithelial cells, upregulating the mRNA expression levels of tight junction genes *ZO1* and *OCLN* (Moosavi et al., 2020). This paracrine mechanism enables *F. prausnitzii* to deliver barrier-enhancing effects without requiring direct contact between live anaerobes and the oxygenated epithelial interface (Pan et al., 2025). Furthermore, the mucus layer composed of mucin 2 (MUC2), secreted by goblet cells, forms the initial barrier protecting the colon from toxins and pathogenic microbes (Liu et al., 2023; Liu Y. et al., 2020). *F. prausnitzii* exerts a marked regulatory effect on maintaining mucus layer homeostasis (Wang et al., 2024). In a sleep-deprivation mouse model, *F. prausnitzii* colonization effectively restored colonic goblet cell counts and rescued MUC2 expression (Wang et al., 2024). Simultaneously, the *F. prausnitzii* metabolite butyrate reinforces the intestinal barrier through dual mechanisms. On one hand, butyrate antagonizes foodborne toxin-induced barrier dysfunction by upregulating tight junction proteins and absorptive cell functional proteins. On the other hand, acting as a signaling molecule, butyrate induces monocyte differentiation into macrophages and promotes the secretion of endogenous antimicrobial proteins like calprotectin, further strengthening the luminal antimicrobial environment (Alberge et al., 2024; Schulthess et al., 2019). Collectively, these findings support a barrier-protective role for *F. prausnitzii* and several of its products in intestinal models. Whether these intestinal effects are sufficient to modify neurological outcomes through the MGBA requires direct experimental validation.

### 3.3 Immunomodulation

Dysfunction of immune cells and inflammatory mediators is central to the progression of neurological diseases (O’Riordan et al., 2025). Imbalanced peripheral T-cell differentiation provides a major immunological foundation for central nervous system (CNS) inflammatory pathology (Bhutta et al., 2024). *F. prausnitzii* modulates the differentiation and phenotype of T cells, B lymphocytes, and dendritic cells (DCs). By influencing the Th17/Treg balance and cytokine-mediated peripheral–central humoral immune crosstalk, it restores immune homeostasis and attenuates central neurodegenerative and inflammatory lesions (Miquel et al., 2013; Bhutta et al., 2024; Han et al., 2025). Studies show that *F. prausnitzii* culture supernatant and its metabolites regulate T-cell subset balance and re-establish immune tolerance (Qiu et al., 2013). *F. prausnitzii* supplementation significantly decreases IL-17-producing Th17 cells in the spleen and increases regulatory T cell (Treg) populations in mesenteric lymph nodes. This suppresses neuroinflammation and alleviates motor deficits (Warren et al., 2026; Song et al., 2016). *F. prausnitzii* also specifically induces a unique regulatory T-cell population in humans: double-positive CD4 + CD8α + (DP8α) T cells. Abundant in the colonic lamina propria, these cells express specific transcription factors and possess both immunosuppressive and cytotoxic functions. Their depletion correlates with disease activity in systemic autoimmune conditions (Louis and Flint, 2009; Foditsch et al., 2014). Furthermore, *F. prausnitzii* reshapes DCs to express high levels of tolerogenic molecules such as IL-10 and IL-27. These DCs suppress pro-inflammatory cytokine production and induce IL-10-secreting anti-inflammatory T cells, forming a defense line that prevents aberrant immune responses from spreading to the CNS (Angelucci et al., 2019; Duncan and Flint, 2008).

*F. prausnitzii* systematically regulates the inflammatory cytokine network, blocking peripheral-to-central inflammatory cascades (Hao et al., 2019; Moon et al., 2023). In a rat model of anxiety and depression, *F. prausnitzii* intervention blocked the abnormal release of corticosterone and C-reactive protein (CRP), prevented rising plasma IL-6 levels, and promoted anti-inflammatory IL-10 release (Hao et al., 2019). In a rheumatoid arthritis model, this bacterium reduced local joint expression of pro-inflammatory cytokines TNF-α, IL-6, and IL-17 (Moon et al., 2023). These results demonstrate that *F. prausnitzii* protects neuronal function by inhibiting pro-inflammatory factors and promoting anti-inflammatory factor secretion, thereby blocking systemic inflammation from activating the BBB and microglia. Additionally, *F. prausnitzii* modulates humoral immunity and host-microbiota–IgA interactions (Gupta et al., 2025). In patients with multiple sclerosis (MS), intestinal *F. prausnitzii* specifically coated by secretory immunoglobulin A (sIgA) is significantly decreased. This reduction weakens peripheral immune tolerance, allowing systemic inflammatory antibodies to cross the BBB (Gupta et al., 2025). In summary, *F. prausnitzii* achieves multidimensional immunomodulation via the MGBA through mechanisms such as balancing T-cell subsets and regulating cytokine networks to intervene in neurological disorders.

## 4 *F. prausnitzii* in neurological and neuropsychiatric disorders

A growing body of human and preclinical evidence has linked alterations in *F. prausnitzii* or the genus *Faecalibacterium* to neurological and neuropsychiatric disorders. However, the strength and nature of this evidence vary substantially across conditions. Human evidence across disorders is heterogeneous in cohort size, age, disease stage, lifestyle, medication use, comorbidities, geographic background, and microbiome profiling methods, limiting cross-study comparability and causal inference. Most human studies are observational; therefore, changes in *F. prausnitzii* abundance should be interpreted as associations rather than evidence of causality. Moreover, animal models cannot fully reproduce the complexity of human exposures and clinical heterogeneity, and preclinical efficacy should therefore not be directly extrapolated to clinical benefit. Table 1 summarizes human studies, Table 2 summarizes relevant preclinical evidence, and Figure 2 provides an overview of the reported disease-specific associations and proposed mechanisms.

**Table 1:** Summary of human clinical studies evaluating *F. prausnitzii* and *Faecalibacterium* in neuropsychiatric and neurological disorders.

Disease	Study design	Population	Detection method	Sample size	Main findings regarding *F. prausnitzii/Faecalibacterium*	Taxonomic level	Key limitations	References
Preclinical AD	Cross-sectional study	Australian	Shotgun metagenomics	19 Aβ High participants and 68 Aβ Low participants	Decreased	Species (*F. prausnitzii*)	Small sample; Cross-sectional	Dissanayaka et al. (2025)
AD	Case–control	Iranian	Real-time PCR	25 AD patients and 20 healthy controls	Decreased	Species (*F. prausnitzii*)	Small sample; Confounding factors	Ahmadi et al. (2026)
PD	Case–control	Iranian	Real-time PCR	25 PD patients and 20 healthy controls	Decreased	Species (*F. prausnitzii*)	Small sample; Confounding factors	Ahmadi et al. (2026)
AD	Case–control	Chinese	Metagenomic sequencing	8 AD patients and 8 normal cognition controls	Decreased	Species (*F. prausnitzii*)	Very small sample; Limited generalizability	Lin et al. (2025)
PD	Case–control	Chinese	16S rRNA sequencing	24 PD patients and 14 healthy controls	Decreased	Species (*F. prausnitzii*)	Small sample; Limited resolution	Li et al. (2017)
AD	Case–control	Japanese	Fecal microbiome analysis	15 MCI, 7 AD patients, and 21 healthy controls	Decreased	Species (*F. prausnitzii*)	Small sample; Confounding factors	Ueda et al. (2021)
PD	Case–control	Indian	16S rRNA sequencing	16 PD patients and 16 healthy controls	Decreased	Species (*F. prausnitzii*)	Small sample; No functional validation	Jacob et al. (2026)
PD	Case–control	Irish	Metagenomics	435 PD patients and 219 healthy controls	Decreased	Species (*F. prausnitzii*)	Confounding factors; Attribution unclear	Hensen and Thiele (2025)
PD	Case–control	Chinese	16S rDNA gene sequencing	64 PDP patients, 36 non-PD patients with pain (nPDP), and 50 healthy controls	Decreased	Species (*F. prausnitzii*)	Cross-sectional; Confounding factors	Yang et al. (2025)
PD & IBD	Case–control	American	Metagenomics	54 PD patients, 26 IBD patients, and 16 healthy controls	Decreased	Species (*F. prausnitzii*)	Attribution unclear; Confounding factors	Krueger et al. (2025)
PD	Case–control	Japanese	Shotgun metagenomics	94 PD patients and 73 healthy controls	Decreased	Species (*F. prausnitzii*)	No functional validation; Confounding factors	Nishiwaki et al. (2024)
ALS	Case–control	Chinese	High-throughput sequencing	6 ALS patients and 5 healthy controls	Decreased	Species (*F. prausnitzii*)	Very small sample; Limited generalizability	Fang et al. (2016)
ALS	Case–control	Chinese	16S rDNA gene sequencing (nasal microbiota)	66 ALS patients and 40 healthy controls	Decreased	Genus (*Faecalibacterium*)	Genus-level analysis; Attribution unclear	Liu et al. (2024)
MS	Cohort	American	16S rRNA gene sequencing	43 MS patients and 42 healthy controls	Decreased	Species (*F. prausnitzii*)	Confounding factors; Attribution unclear	Gupta et al. (2025)
MS	Case–control	International Multiple Sclerosis Microbiome Study	16S rRNA/Shotgun sequencing	576 MS patients and 1,152 healthy controls	Decreased	Species (*F. prausnitzii*)	Geographic heterogeneity; Confounding factors	iMSMS Consortium (2022)
MS	Case–control	Danish	Shotgun metagenomics	148 MS and 148 healthy controls	Decreased	Species (*F. prausnitzii*)	Confounding factors; No causal inference	Thirion et al. (2023)
MS	Cohort	Romania	16S rRNA gene sequencing	39 MS patients and 21 healthy controls	Decreased	Species (*F. prausnitzii*)	Small sample; Limited generalizability	Paraschiv et al. (2024)
MS	Cohort	German	16S rRNA gene sequencing	25 MS patients and 14 healthy controls	Decreased	Species (*F. prausnitzii*)	Small sample; Observational	Swidsinski et al. (2017)
Stroke	Case–control	Chinese	16S rDNA V3-V4 sequencing	59 Stroke patients and 31 healthy controls	Decreased	Species (*F. prausnitzii*)	Cross-sectional; Confounding factors	Luo et al. (2024)
ADHD	Case–control	Chinese	Shotgun metagenomics	94 ADHD patients and 94 age- and gender-matched controls	Decreased	Genus (*Faecalibacterium*)	Genus-level analysis; Confounding factors	Wang et al. (2025)
ADHD	Case–control	Chinese	High-throughput pyrosequencing	51 ADHD patients and 32 healthy controls	Decreased	Genus (*Faecalibacterium*)	Small sample; Limited resolution	Jiang et al. (2018)
ADHD	Case–control	Chinese	Shotgun metagenomics	17 ADHD patients and 17 healthy controls	Decreased	Species (*F. prausnitzii*)	Small sample; Observational	Wan et al. (2020)
ASD	Case–control	American	16S rRNA gene sequencing	21 ASD children and 23 neurotypical controls	Decreased	Species (*F. prausnitzii*)	Small sample; Confounding factors	Kang et al. (2018)
ASD	Case–control	Uruguayan	16S rRNA gene sequencing	30 ASD children and 28 typical development controls	Decreased	Species (*F. prausnitzii*)	Small sample; Cross-sectional	Mendive Dubourdieu and Guerendiain (2023)
ASD	Cohort	Chinese	16S rRNA gene sequencing	58 ASD children and 30 typical development controls	Increased	Species (*F. prausnitzii*)	Conflicting findings; Geographic heterogeneity	Huang et al. (2025)
ASD	Case–control	Italian	16S rRNA gene sequencing	11 ASD children and 14 typical development controls	Increased	Species (*F. prausnitzii*)	Very small sample; Limited generalizability	Coretti et al. (2018)

Aβ, Amyloid-beta; AD, Alzheimer’s Disease; ADHD, Attention-Deficit/Hyperactivity Disorder; AIS, Acute Ischemic Stroke; ALS, Amyotrophic Lateral Sclerosis; ASD, Autism Spectrum Disorder; *F. prausnitzii, Faecalibacterium prausnitzii*; IBD, Inflammatory Bowel Disease; MCI, Mild Cognitive Impairment; MS, Multiple Sclerosis; nPDP, non-Parkinson’s Disease with Pain; PCR, Polymerase Chain Reaction; PD, Parkinson’s Disease; PDP, Parkinson’s Disease with Pain; rDNA, ribosomal Deoxyribonucleic Acid; rRNA,

**Table 2:** Summary of preclinical studies linking *F. prausnitzii* or the genus *Faecalibacterium* to neurological outcomes.

Disease	Animal model	Intervention/strain	Detection methods	Major findings	Evidence interpretation	Key limitations	References
AD	Aβ25–35-induced AD mice (ddY mice)	Live Fp360; pasteurized Fp14; live Fp14; Fp14 supernatant	Behavioral tests; molecular assays; microbiome sequencing	Improved memory function; reduced oxidative stress and mitochondrial dysfunction; effects differed by strain and viability	Direct strain-specific evidence	Animal model; no human validation	Ueda et al. (2021)
AD	APP/PS1 transgenic mice	Tibetan fermented milk supplementation	Behavioral tests; molecular assays; microbiome sequencing	Improved cognition; reduced Aβ accumulation and neurodegeneration; increased *Faecalibacterium* abundance	Indirect microbiota evidence	Multicomponent intervention; attribution unclear	Liu et al. (2020a)
AD	APP/PS1 transgenic mice (young vs. middle-aged)	Voluntary wheel running	Behavioral tests; molecular assays; metabolomics; microbiome sequencing	Improved cognition and reduced neuroinflammation only in young mice; age-dependent microbiome changes	Indirect microbiota evidence	No direct *F. prausnitzii* administration; attribution unclear	Wang et al. (2021)
PD	α-synuclein-overexpressing (Thy1-ASO) mice	Oral *F. prausnitzii* or microbial consortium	Microbiome analysis; motor/GI assessment; immune assays; α-synuclein analysis	Improved motor and gastrointestinal deficits; reduced brain α-synuclein aggregation; enhanced anti-inflammatory responses	Direct strain-specific evidence	Animal model; limited clinical translation	Moiseyenko et al. (2026)
PD	PC12/Caco-2 gut–brain axis co-culture model	*F. prausnitzii* + *L. plantarum* + Herba Patriniae extract	Metabolite analysis; inflammatory assays; mitochondrial assays; barrier integrity assays	Reduced oxidative stress and inflammation; increased butyrate; improved mitochondrial and barrier function	Multicomponent mechanistic evidence	Multicomponent intervention; attribution unclear	Wu et al. (2026)
PD	AAV-α-synuclein rat model	Voluntary exercise	Histology; neurochemistry; microbiome and bile acid analysis	Altered gut microbiota and bile acid metabolism; reduced intestinal *Faecalibacterium* abundance	Indirect microbiota evidence	No direct bacterial intervention; causality unclear	Leclair-Visonneau et al. (2020)
Ischemic stroke	MCAO mouse model	Synbiotic formulation containing *F. prausnitzii*-related intervention	Behavioral tests; histology; molecular assays; 16S sequencing	Reduced neurological deficits, infarct volume, inflammation, and neuronal death; increased *F. prausnitzii* abundance	Multistrain/microbiota intervention evidence	Attribution unclear; species-specific effect uncertain	Kim et al. (2024)
Ischemic stroke	Aged MCAO mice	Young FMT or SCFA-producing bacterial consortium (*B. longum*, *C. symbiosum*, *F. prausnitzii*, *L. fermentum*)	Behavioral tests; inflammation assays; microbiome sequencing; metabolomics; SCFA measurement	Improved post-stroke recovery; increased SCFA levels; reduced inflammatory responses	Multistrain microbiota evidence	Multispecies intervention; *F. prausnitzii*-specific contribution unclear	Lee et al. (2020)

AAV, adeno-associated virus; AD, Alzheimer’s disease; Aβ, amyloid-beta; APP/PS1, amyloid precursor protein/presenilin-1; *B. longum, Bifidobacterium longum; C. symbiosum, Clostridium symbiosum*; Caco-2, human colorectal adenocarcinoma cells; ENS, enteric nervous system; *F. prausnitzii, Faecalibacterium prausnitzii*; FMT, fecal microbiota transplantation; Fp14, *Faecalibacterium prausnitzii* strain 14; Fp360, *Faecalibacterium prausnitzii* strain 360; GI, gastrointestinal; GBA, gut–brain axis; GFAP, glial fibrillary acidic protein; GSK3β, glycogen synthase kinase 3 beta; HP, Herba Patriniae; i.c.v., intracerebroventricular; i.g., intragastric; *L. fermentum*, *Limosilactobacillus fermentum*; *L. plantarum*, Lactiplantibacillus plantarum; MCAO, middle cerebral artery occlusion; PD, Parkinson’s disease; PA, passive avoidance; PC12, rat pheochromocytoma cells; SCFA, short-chain fatty acid; TMAO, trimethylamine N-oxide; ZO-1, zonula occludens-1.

**Figure 2:**
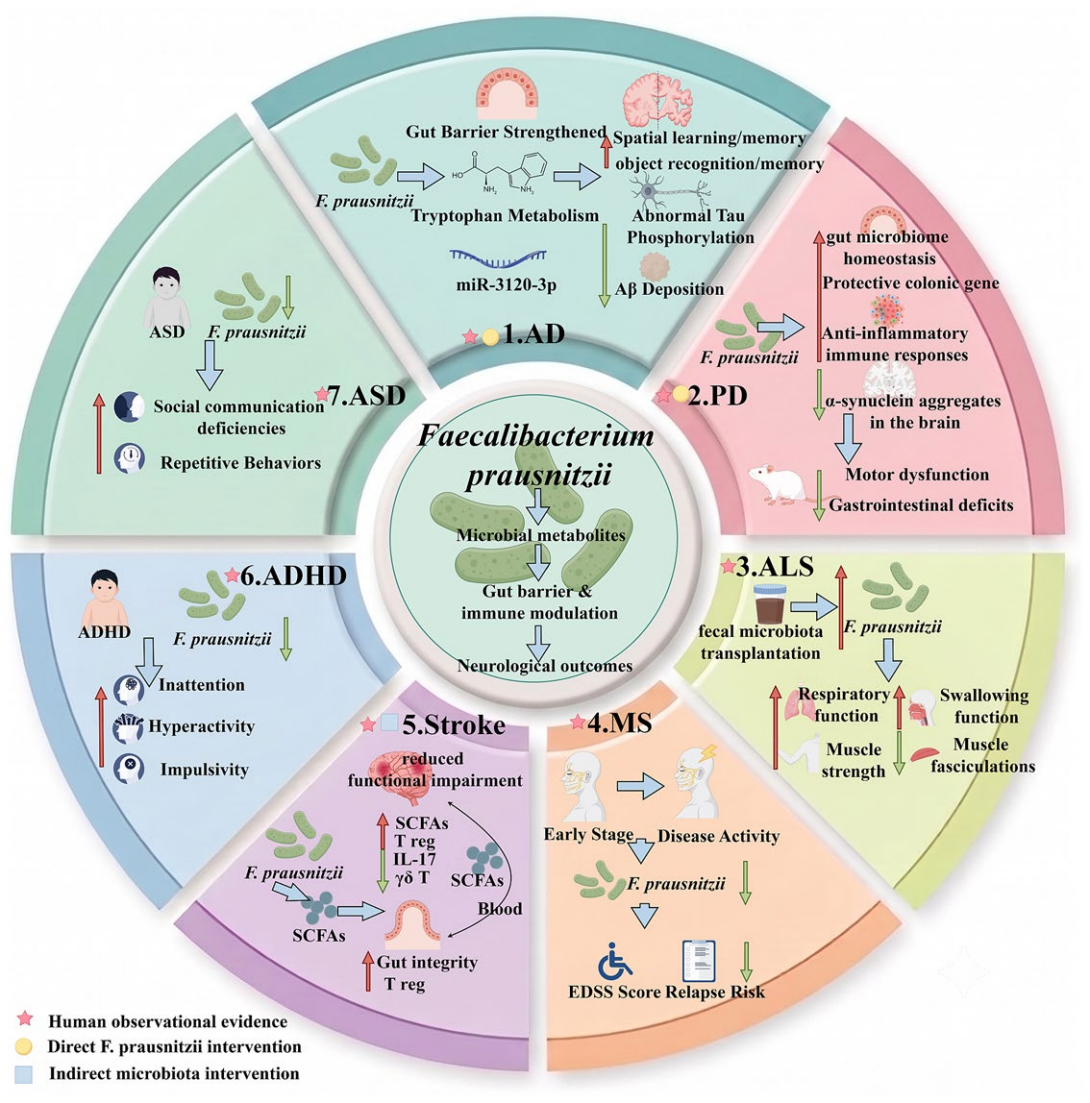
Reported associations, preclinical effects, and proposed roles of *Faecalibacterium prausnitzii* across neurological and neuropsychiatric disorders. Aβ, amyloid-beta; AD, Alzheimer’s disease; ADHD, attention-deficit/hyperactivity disorder; ALS, amyotrophic lateral sclerosis; ASD, autism spectrum disorder; EDSS, Expanded Disability Status Scale; IL-17, interleukin-17; miR-3120-3p, microRNA-3120-3p; MS, multiple sclerosis; PD, Parkinson’s disease; SCFAs, short-chain fatty acids; Treg, regulatory T cells; γδT, gamma delta T cells.

### 4.1 *F. prausnitzii* and Alzheimer’s disease

#### 4.1.1 Human evidence

AD is a neurodegenerative condition with both familial and sporadic forms. It is biologically characterized by amyloid-beta (Aβ) plaques and tau neurofibrillary tangles, typically presenting as amnestic cognitive impairment (Knopman et al., 2021). Multiple studies have reported associations between altered *F. prausnitzii* abundance and AD-related clinical or biological features. The abundance of this bacterium declines significantly with worsening cognitive impairment (Dissanayaka et al., 2025; Ahmadi et al., 2026; Lin et al., 2025; Li et al., 2017). Even in cognitively unimpaired individuals, its abundance correlates with cerebral Aβ deposition (Dissanayaka et al., 2025). In a cross-sectional study, gut *F. prausnitzii* abundance correlated positively with Japanese version of the Montreal Cognitive Assessment (MoCA-J) scores. Patients with mild cognitive impairment (MCI) exhibited a significant reduction in gut *F. prausnitzii* compared with healthy controls (Ueda et al., 2021). A study in older adults in China noted that gut *Faecalibacterium* abundance in MCI patients was approximately 30% lower than in healthy controls, displaying a positive correlation with cognitive scores (Zhang et al., 2021). Multi-omics analysis confirmed that beneficial bacteria, represented by *F. prausnitzii*, were markedly depleted in AD patients. This depletion correlated with elevated intestinal inflammatory markers, specifically calprotectin and zonulin (Ahmadi et al., 2026). Cai et al. also revealed complex signaling between *F. prausnitzii* and host miRNAs, finding that *F. prausnitzii* abundance was closely linked to plasma exosomal miR-3120-3p levels in AD patients (Lin et al., 2025). Based on these associations, clinical interventions have aimed to improve cognition by increasing *F. prausnitzii* abundance. A 12-week yoga intervention increased *F. prausnitzii* abundance and improved cognitive scores in AD patients (Tiwari et al., 2026). Additionally, probiotic supplementation to elevate *F. prausnitzii* levels strengthened intestinal barrier function and modulated the tryptophan metabolic axis in AD patients. This suggests that the bacterium may attenuate AD progression by regulating systemic immune activation and metabolic balance (Leblhuber et al., 2018). Together, these findings suggest that reduced *F. prausnitzii* abundance is linked to AD-related cognitive impairment, although whether this alteration contributes to disease progression or represents a consequence of disease remains unclear.

#### 4.1.2 Preclinical evidence

Preclinical intervention studies provide more direct evidence for the potential neuroprotective effects of *F. prausnitzii* in experimental AD models. Takuji et al. isolated multiple *F. prausnitzii* strains from healthy human feces and evaluated their effects in an Aβ-induced AD mouse model (Ueda et al., 2021). The interventions showed clear strain-specific differences: pasteurized *F. prausnitzii* 14 (Fp14) significantly improved cognition, whereas its live form showed little effect (Ueda et al., 2021); conversely, *F. prausnitzii* 360 (Fp360) required live administration to mitigate spatial and long-term memory deficits (Ueda et al., 2021). These contrasting responses indicate that the neurological effects of *F. prausnitzii* are strain- and viability-dependent, and that species-level abundance alone may not predict the functional or therapeutic effects of individual strains. In APP/PS1 transgenic mice, Tibetan fermented milk supplementation increased the relative abundance of *Faecalibacterium*, improved spatial learning and object recognition, and reduced Aβ deposition in the cortex and hippocampus (Liu J. et al., 2020). In the same model, voluntary wheel running showed age-dependent effects: when initiated at 9 weeks of age, it attenuated tau phosphorylation and neuroinflammation, enhanced synaptic plasticity-related proteins, and improved learning and memory, whereas initiation at 24 weeks of age failed to improve cognition and was accompanied by a reduction in *Faecalibacterium* abundance (Wang et al., 2021). Collectively, direct strain-administration studies support strain- and viability-dependent effects, whereas fermented milk and exercise studies provide more indirect evidence because they modify the broader gut microbiota. Thus, the specific causal contribution of *F. prausnitzii* to AD remains to be established through further species-specific and clinical validation.

### 4.2 *F. prausnitzii* and Parkinson’s disease

#### 4.2.1 Human evidence

PD is a progressive neurodegenerative disorder characterized pathologically by loss of substantia nigra neurons and α-synuclein aggregation. Clinically, it presents with rigidity, resting tremor, and gait impairment (Morris et al., 2024). Clinical studies demonstrate that the abundance of *F. prausnitzii* and its metabolites are significantly reduced in PD patients, correlating closely with disease severity (Ahmadi et al., 2026; Jacob et al., 2026; Hensen and Thiele, 2025; Yang et al., 2025; Krueger et al., 2025; Nishiwaki et al., 2024). A study in an Indian cohort found that despite distinct geographical and dietary backgrounds, *F. prausnitzii* abundance and network centrality served as key markers distinguishing PD patients from healthy controls (Jacob et al., 2026). Another study utilizing constraint-based metabolic modeling across 435 PD patients and 219 healthy individuals showed that reduced relative abundance of *F. prausnitzii* was closely linked to impaired host capacity to synthesize butyrate, myristate, and pantothenate (Hensen and Thiele, 2025). Combined 16S rDNA sequencing and LC–MS analysis revealed distinct gut microbial signatures in PD patients with pain, centered on *F. prausnitzii* abundance fluctuations. A strong negative correlation was observed between *F. prausnitzii* abundance and plasma lysophosphatidic acid (LysoPA) levels (Yang et al., 2025). These results suggest *F. prausnitzii* contributes to PD pathogenesis by influencing metabolic outputs. Conversely, increasing *F. prausnitzii* abundance through diet or probiotics alleviates PD symptoms. For instance, the Mediterranean diet specifically increases *F. prausnitzii* and its metabolite butyrate. These metabolites participate in synaptic plasticity and inflammatory regulation, underpinning improvements in memory, attention, and executive functions (Ibeas-Perez et al., 2026). Pellecchia et al. administered a 12-week synbiotic treatment to PD patients, observing a significant increase in gut *F. prausnitzii* abundance. This microbial shift was closely associated with relief of non-motor symptoms, including depression, anxiety, and autonomic dysfunction (Andreozzi et al., 2024). These clinical findings support an association between *F. prausnitzii* abundance and PD-related features; however, current evidence is insufficient to establish a protective role or clinical biomarker utility.

#### 4.2.2 Preclinical evidence

Preclinical models provide more direct evidence for potential protective effects of *F. prausnitzii* in PD. Anastasiya et al. demonstrated that supplementing *F. prausnitzii* in α-synuclein-overexpressing Thy1-ASO mice significantly corrected gut dysbiosis, exerted anti-inflammatory effects, protected the gastrointestinal tract, and improved motor deficits. Crucially, it downregulated pathological α-synuclein aggregation in the brain (Moiseyenko et al., 2026). Wu et al. used an *F. prausnitzii*-containing synbiotic formulation to target the 5-HTP–TMAO metabolic axis. This formulation exerted direct neuroprotective effects by raising butyrate levels, inhibiting trimethylamine N-oxide (TMAO) production, and correcting abnormal 5-hydroxytryptophan (5-HTP) uptake (Wu et al., 2026). In rats with adeno-associated virus (AAV)-induced α-synuclein overexpression in the substantia nigra, researchers observed a significant reduction in intestinal *Faecalibacterium* levels, mirroring the gut dysbiosis phenotype observed in PD patients (Leclair-Visonneau et al., 2020). Collectively, these studies support a potential therapeutic role for direct *F. prausnitzii* administration in selected PD models, while evidence from synbiotic formulations and disease-associated microbiome changes remains indirect.

### 4.3 *F. prausnitzii* and amyotrophic lateral sclerosis

#### 4.3.1 Human evidence

Amyotrophic lateral sclerosis (ALS) is a neurodegenerative disorder characterized by progressive, painless muscle weakness caused by motor neuron loss in the brain, brainstem, and spinal cord (Leclair-Visonneau et al., 2020). Multiple clinical studies report marked gut dysbiosis in ALS patients, with reduced *F. prausnitzii* abundance as a consistently recurring feature that correlates with disease progression and severity (Fang et al., 2016; Gerhardt and Mohajeri, 2018). Although heterogeneity exists across cohorts due to diet, lifestyle, and disease subtypes, this link has been validated across several studies (Fang et al., 2016; Gerhardt and Mohajeri, 2018). Notably, the role of *F. prausnitzii* in ALS may extend beyond the gastrointestinal tract. A study examining the nasal microbiome of ALS patients revealed that nasal *Faecalibacterium* abundance correlated positively with revised ALS Functional Rating Scale (ALSFRS-R) scores (Liu et al., 2024). However, given that *Faecalibacterium* is an extremely oxygen-sensitive obligate anaerobe, its detection in the nasal mucosa may reflect microbial translocation or systemic immune alterations rather than active respiratory colonization, warranting cautious interpretation. A recent case report showed that two advanced ALS patients with respiratory failure experienced significant improvements in respiratory function, muscle strength, and motor capability following FMT. This was accompanied by increased intestinal *F. prausnitzii* abundance and elevated levels of arginine biosynthesis metabolites, suggesting that *F. prausnitzii* may contribute to reversing respiratory failure through metabolic regulation (Yan et al., 2024). However, in a larger randomized controlled trial (RCT) by Feng et al., FMT reshaped gut microbiota structure but failed to achieve statistically significant slowing of ALSFRS-R score decline (Feng et al., 2024). Discrepancies between these studies may stem from differences in sample size and patient inclusion criteria. These findings imply that boosting beneficial bacteria like *F. prausnitzii* may primarily alleviate comorbid symptoms—such as respiratory dysfunction, constipation, and psychological distress—rather than fundamentally reversing neurodegeneration.

### 4.4 *F. prausnitzii* and multiple sclerosis

#### 4.4.1 Human evidence

MS is an autoimmune disorder of the central nervous system. Its primary pathological hallmark is autoreactive T cell targeting of myelin antigens, resulting in demyelination and neurological disability (Dendrou et al., 2015). Depletion of *F. prausnitzii* represents a central feature of MS-associated gut dysbiosis (Gupta et al., 2025; iMSMS Consortium, 2022; Thirion et al., 2023; Paraschiv et al., 2024; Mokhtarzade et al., 2022; Mokhtarzade et al., 2021; Swidsinski et al., 2017). In an International MS Microbiome Study comprising 576 MS patients and 1,152 healthy controls, untreated MS patients exhibited a marked decline in *F. prausnitzii* abundance. This reduction was more pronounced in progressive MS than in relapsing–remitting MS, indicating that loss of this bacterium correlates closely with disease progression and severity (iMSMS Consortium, 2022). Although MS patients reported higher diet quality scores (including greater intake of fruits and vegetables that foster bacterial growth), low *F. prausnitzii* levels persisted. This discrepancy suggests that the depletion is driven by the MS disease state rather than environmental dietary confounders (iMSMS Consortium, 2022). Similarly, metagenomic sequencing by Thirion et al. in a Danish cohort of 148 MS patients and 148 matched healthy controls revealed significantly higher *F. prausnitzii* abundance during non-active disease phases (Thirion et al., 2023). In a 2-month prospective longitudinal study, Paraschiv et al. confirmed reduced gut *F. prausnitzii* in MS patients (Paraschiv et al., 2024). Although disease-modifying therapies (DMTs) and IgY supplementation altered other beneficial taxa, they failed to reverse the reduction in *F. prausnitzii*, reinforcing its deficit as a stable hallmark of MS-related dysbiosis (Paraschiv et al., 2024). Regarding lifestyle interventions, Mokhtarzade et al. showed that lifelong exercise helps maintain levels of probiotics such as *F. prausnitzii*, which confers clinical protection for brain parenchymal volume in MS patients (Mokhtarzade et al., 2022). However, another study by the same group revealed that a short-term home-based exercise program significantly improved anxiety and depression in MS patients without altering *F. prausnitzii* abundance. This suggests the bacterium responds to long-term lifestyle habits rather than short-term exercise (Mokhtarzade et al., 2021). Furthermore, Swidsinski et al. identified *F. prausnitzii* as one of the most severely depleted bacteria in MS patients, yet a 6-month ketogenic diet effectively restored its levels to baseline or higher (Swidsinski et al., 2017). These findings underscore the importance of *F. prausnitzii* in MS pathophysiology. Nevertheless, current evidence derives mainly from clinical cross-sectional and longitudinal observations. Functional validation in animal models is necessary to clarify its causal role and specific immunomodulatory pathways in MS.

### 4.5 *F. prausnitzii* and stroke

#### 4.5.1 Human evidence

Stroke is a leading cause of mortality and disability worldwide, presenting a major public health challenge. Clinically, stroke is categorized as ischemic stroke, caused by vascular occlusion, or hemorrhagic stroke, caused by vascular rupture (Hankey, 2014). Emerging evidence indicates that stroke impairs intestinal function, increases gut permeability, activates intestinal immune cells, and drives gut microbiota translocation (Stanley et al., 2018). A positive feedback loop is increasingly recognized: post-stroke brain changes trigger gut dysbiosis and inflammation, which subsequently exacerbate central neuroinflammation and stroke progression (Benakis et al., 2016). In a case–control study of 59 patients with acute ischemic stroke and 31 age-matched healthy controls, Luo et al. found that *F. prausnitzii* abundance correlated negatively with stroke severity, adverse prognostic indicators, and inflammatory markers, suggesting that *F. prausnitzii* warrants further investigation as a candidate microbial biomarker of acute ischemic stroke severity and prognosis (Luo et al., 2024).

#### 4.5.2 Preclinical evidence

In animal models, modulating the gut microbiome via FMT or probiotic supplementation before or during stroke significantly alters recovery outcomes (Rahman et al., 2024; Lee et al., 2020). Lee et al. observed that transplanting healthy human feces into middle cerebral artery occlusion (MCAO) mice markedly improved stroke outcomes (Lee et al., 2020). Further investigations confirmed that transplanting specific SCFA-producing strains, particularly *F. prausnitzii*, elevated SCFA levels in host gut, blood, and brain tissues, thereby attenuating neurological deficits and inflammatory responses. These findings suggest that *F. prausnitzii* promotes stroke recovery by elevating SCFA concentrations along the gut–blood–brain axis (Lee et al., 2020). Additionally, Rahman et al. demonstrated in an MCAO rat model that a multi-strain synbiotic intervention significantly alleviated post-stroke neuroinflammation and restored intestinal barrier function, accompanied by a marked recovery in intestinal *F. prausnitzii* abundance (Rahman et al., 2024). Overall, human and animal studies implicate SCFA-producing gut microbiota, including *F. prausnitzii*, in post-stroke inflammatory and metabolic responses. However, current evidence does not establish *F. prausnitzii* as an independent regulator of stroke recovery.

### 4.6 *F. prausnitzii* and attention-deficit/hyperactivity disorder

#### 4.6.1 Human evidence

Attention-deficit/hyperactivity disorder (ADHD) is a neurodevelopmental disorder characterized by persistent, pervasive inattention and/or hyperactivity-impulsivity inconsistent with developmental level, leading to functional impairment (Koirala et al., 2024). Its etiology involves genetic, environmental, and physiological factors. The neurodevelopmental biology of ADHD remains unclear, and reliable diagnostic biomarkers are lacking, leaving diagnosis reliant on behavioral evaluations (Koirala et al., 2024). Evidence links gut microbial profiles with ADHD symptom severity, suggesting that microbes influence neurotransmitter function via metabolites like SCFAs; however, causality and specific mechanisms remain unclarified (Wang et al., 2023; Wang et al., 2025; Ast et al., 2025; Bundgaard-Nielsen et al., 2023). Wang et al. used 16S rRNA sequencing to show that children with ADHD had significantly higher gut microbial diversity (Shannon and Chao indices) than controls (Wang et al., 2020). In a study by Jiang et al., high-throughput pyrosequencing of fecal samples from 51 children with ADHD and 32 healthy controls revealed a significant reduction in relative *Faecalibacterium* abundance in the ADHD group (Jiang et al., 2018). Further analysis demonstrated that *Faecalibacterium* abundance correlated negatively with parent-reported ADHD symptom severity, highlighting its potential as a microbial biomarker (Jiang et al., 2018). Utilizing metagenomic sequencing, Wan et al. confirmed a marked decrease in *F. prausnitzii* in ADHD patients, revealing significant microbial differences in dopamine and 5-hydroxytryptophan (5-HTP) metabolic pathways (Wan et al., 2020). Although reduced *F. prausnitzii* is consistently observed in clinical studies, the precise mechanisms by which it influences neurodevelopment remain to be elucidated. Moreover, preclinical intervention studies that directly establish a causal role for *F. prausnitzii* in ADHD are still lacking. Nevertheless, targeted microbiota-based interventions may offer novel perspectives for the clinical diagnosis and personalized treatment of ADHD.

### 4.7 *F. prausnitzii* and autism spectrum disorder

#### 4.7.1 Human evidence

Autism spectrum disorder (ASD) is a highly heterogeneous and etiologically complex neurodevelopmental condition characterized by impaired social interaction and communication alongside repetitive, stereotyped behaviors (Fattorusso et al., 2019). Epidemiological data show that children with ASD experience a significantly higher rate of gastrointestinal issues than neurotypical peers, with approximately 40% presenting with overt dysfunction such as constipation, diarrhea, and abdominal pain (Wang et al., 2011; Fulceri et al., 2016). Recent studies suggest that these gastrointestinal disturbances and central nervous system symptoms may stem from an inflammatory state induced by gut dysbiosis, pointing to gut–brain interactions as a pivotal player in ASD pathogenesis (Wang et al., 2011; Fulceri et al., 2016; Navarro et al., 2016). Fluctuations in *F. prausnitzii* in ASD have attracted considerable attention, though findings vary geographically (Kang et al., 2018; Mendive Dubourdieu and Guerendiain, 2023; Huang et al., 2025). In a US study of 21 children with ASD, Kang et al. reported lower gut microbial diversity and significantly reduced *F. prausnitzii* abundance compared to neurotypical controls (Kang et al., 2018). Similarly, a study evaluating ASD children with different nutritional statuses found that those with overweight or obesity had significantly lower *F. prausnitzii* abundance than BMI-matched healthy controls (Mendive Dubourdieu and Guerendiain, 2023). Conversely, a cohort study in Guangxi, China, demonstrated a marked enrichment of *F. prausnitzii* in children with ASD, with taxonomic changes correlating positively with arginine/proline metabolic pathways (Huang et al., 2025). Coretti et al. similarly observed elevated *F. prausnitzii* levels in young children (aged 2–4 years) with ASD in Naples, linking high abundance to increased fecal butyrate concentrations and enriched metabolic pathways (Coretti et al., 2018). The direction of the association between *F. prausnitzii* abundance and ASD remains unresolved, likely due to cohort heterogeneity in age, diet, nutritional status, geography, sample size, and microbiome profiling methods, including differences in sequencing platforms and taxonomic resolution (Huang et al., 2025). Dietary interventions and microbial modulation provide fresh possibilities for ASD management (Raghavan et al., 2023; Grimaldi et al., 2017; Grimaldi et al., 2018). Raghavan et al. showed that administering β-1,3/1,6-glucan to ASD patients effectively increased gut probiotics like *F. prausnitzii*, improving behavior and sleep quality (Raghavan et al., 2023). Grimaldi et al. used an *in vitro* gut model to evaluate fecal fermentation responses to bimuno-galactooligosaccharide (B-GOS) prebiotics in ASD and non-ASD children. B-GOS intervention significantly elevated *F. prausnitzii* abundance and altered fecal ethanol and lactate levels (Grimaldi et al., 2017). In a subsequent clinical study of 30 children with ASD, Grimaldi et al. evaluated an exclusion diet (such as gluten-free or casein-free)(Grimaldi et al., 2018). Children adhering to the diet showed reduced abdominal pain and bowel dysfunction scores, alongside increased gut *F. prausnitzii* abundance (Grimaldi et al., 2018). Together, these studies indicate that shifts in *F. prausnitzii* abundance correlate with symptom relief in ASD. Given the inconsistent direction of association across cohorts, the utility of *F. prausnitzii* as an ASD biomarker remains uncertain, and larger multicenter longitudinal studies with standardized microbiome profiling are needed to clarify its relationship with ASD phenotypes.

## 5 Future perspectives and concluding remarks

Gut dysbiosis is closely linked to the pathogenesis and progression of various CNS disorders. Clinically, reduced abundance of *F. prausnitzii* has emerged as a hallmark intestinal alteration across several neurological conditions, including AD, PD, ALS, and MS. In animal models, *F. prausnitzii* exerts multifaceted protective effects in the bidirectional regulation of the MGBA. It maintains gastrointestinal mucosal barrier integrity, reduces mucosal permeability, and downregulates peripheral pro-inflammatory cytokines, thereby preventing inflammatory cascades from spreading to the central nervous system. These two evidence streams should be interpreted separately, because associations observed in humans do not establish that depletion of *F. prausnitzii* contributes causally to disease development or progression. Collectively, these findings support *F. prausnitzii* as a potentially relevant microbial component of the MGBA, although its causal contribution and therapeutic significance in humans remain to be established.

The strength and type of evidence differ markedly across disorders. AD and PD include both human association studies and preclinical intervention evidence; MS is supported by relatively large human cohorts but lacks comparable species-specific intervention data; stroke evidence remains limited and partly derives from FMT or SCFA-producing bacterial consortia; ALS and ADHD are mainly supported by observational evidence; and findings in ASD remain directionally inconsistent. Importantly, clinical intervention studies directly evaluating oral *F. prausnitzii* formulations in patients with neurological disorders are still lacking. Human studies therefore mainly support associations, while improvements observed after exercise, dietary interventions, prebiotics, synbiotics, or FMT cannot be attributed specifically to *F. prausnitzii*, because these interventions simultaneously affect the broader gut microbiota and host physiology. Moreover, beneficial clinical outcomes may occur without a parallel increase in *F. prausnitzii*, indicating that changes in its abundance should not be regarded as a universal surrogate of therapeutic response. In contrast, direct administration or colonization with specific *F. prausnitzii* strains in animal models provides more direct evidence for strain- and model-dependent neuroprotective effects. In AD models, it improves spatial and long-term memory while reducing Aβ deposition and abnormal tau phosphorylation. In PD models, it elevates striatal histone acetylation levels, protects dopaminergic neurons, and downregulates pathological α-synuclein aggregation in the brain. In MCAO models, it effectively raises central SCFA concentrations and alleviates neurological deficits.

Mechanistically, current *in vitro* and *in vivo* studies highlight several key pathways, although the specificity of this evidence varies. Findings involving MAM, EVs, or direct *F. prausnitzii* administration provide relatively species-linked evidence, whereas many observations involving butyrate, SCFA signaling, HDAC inhibition, and downstream inflammatory pathways represent biologically plausible but indirect support. First, *F. prausnitzii* utilizes acetate to produce its core metabolite, butyrate. Butyrate reinforces tight junctions via the AKT/AMPK pathway and exerts HDAC inhibitory activity to restore impaired neuronal gene transcription, promoting neurogenesis and synaptic plasticity. Second, *F. prausnitzii* secretes MAM and EVs to block NF-κB activation in host epithelial cells. It also restores colonic goblet cell counts and MUC2 expression, thereby reinforcing the mucus barrier. Third, through the synthesis of phenolic compounds, it activates the AKT/Nrf2 signaling pathway to suppress microglial overactivation and oxidative stress. Finally, *F. prausnitzii* re-establishes immune tolerance by modulating the Th17/Treg balance and inducing double-positive CD4^+^CD8α^+^ T cells. It upregulates tolerogenic molecules (IL-10 and IL-27) and systematically downregulates pro-inflammatory markers, including IL-6, IL-17, TNF-α, CRP, and TMAO, while promoting anti-inflammatory IL-10 release.

Several barriers must be addressed before the clinical translation of *F. prausnitzii*. Its marked strain specificity and extreme oxygen sensitivity raise important challenges related to strain selection, formulation, storage stability, viable dose, delivery, colonization, and patient-level safety. Future studies should prioritize strain-specific characterization and mechanistic validation, together with longitudinal human cohorts using standardized microbiome profiling and adequate control of major confounders. Integrating biochemical metabolite profiling, dietary tracking, and multi-omics approaches may further clarify how *F. prausnitzii* interacts with host metabolic, immune, and neurobiological pathways over time. Ultimately, large-scale, multicenter randomized controlled trials with long-term follow-up are required to establish its safety, feasibility, colonization efficiency, and therapeutic efficacy in neurological populations. Overall, *F. prausnitzii* represents a biologically plausible and translationally interesting candidate within the MGBA, but its clinical therapeutic value remains to be established.
